# Ontogeny of CX3CR1-EGFP expressing cells unveil microglia as an integral component of the postnatal subventricular zone

**DOI:** 10.3389/fncel.2015.00037

**Published:** 2015-02-17

**Authors:** Anna L. Xavier, Flavia R. S. Lima, Maiken Nedergaard, João R. L. Menezes

**Affiliations:** ^1^Programa em Ciências Morfológicas, Programa de Diferenciação Celular, Laboratório de Neuroanatomia Celular, Instituto de Ciências Biomédicas, Centro de Ciências da Saúde, Universidade Federal do Rio de JaneiroRio de Janeiro, Brazil; ^2^Center for Translational Neuromedicine, University of Rochester Medical SchoolRochester, NY, USA; ^3^Laboratório de Morfogênese Celular, Instituto de Ciências Biomédicas, Centro de Ciências da Saúde, Universidade Federal do Rio de JaneiroRio de Janeiro, Brazil

**Keywords:** microglia, CX3CR1, subventricular zone, rostral migratory stream, neurogenesis

## Abstract

The full spectrum of cellular interactions within CNS neurogenic niches is still poorly understood. Only recently has the monocyte counterpart of the nervous system, the microglial cells, been described as an integral cellular component of neurogenic niches. The present study sought to characterize the microglia population in the early postnatal subventricular zone (SVZ), the major site of postnatal neurogenesis, as well as in its anterior extension, the rostral migratory stream (RMS), a pathway for neuroblasts during their transit toward the olfactory bulb (OB) layers. Here we show that microglia within the SVZ/RMS pathway are not revealed by phenotypic markers that characterize microglia in other regions. Analysis of the transgenic mice strain that has one locus of the constitutively expressed fractalkine CX3CR1 receptor replaced by the gene encoding the enhanced green fluorescent protein (EGFP) circumvented the antigenic plasticity of the microglia, thus allowing us to depict microglia within the SVZ/RMS pathway during early development. Notably, microglia within the early SVZ/RMS are not proliferative and display a protracted development, retaining a more immature morphology than their counterparts outside germinal layers. Furthermore, microglia contact and phagocyte radial glia cells (RG) processes, thereby playing a role on the astroglial transformation that putative stem cells within the SVZ niche undergo during the first postnatal days.

## Introduction

Most often neurogenesis occurs in discrete regions known as germinal or germinative zones (Götz and Huttner, 2005; Franco and Müller, 2013). Interactions of specific cellular and molecular components of the neurogenic niche determine the progeny output (Jones and Wagers, 2008; Pathania et al., 2010; Lim and Alvarez-Buylla, 2014). In postnatal germinal zones, such as the adult telencephalic subventricular zone (SVZ) (Lim and Alvarez-Buylla, 2014) and subgranular layer of the hippocampus dentate gyrus (Seri et al., 2004), a common set of cells with distinct features are observed (Miller and Gauthier-Fisher, 2009), including quiescent multipotent neural stem cell with astrocytic characteristics, support cells, intermediary progenitors, immediate progeny, blood vessels and a specialized extracellular matrix (Tavazoie et al., 2008; Miller and Gauthier-Fisher, 2009). In the last few years it has been demonstrated that the monocyte counterpart of the nervous system, the microglial cell, is a full component of neurogenic niches (Mercier et al., 2002; Sierra et al., 2010; Olah et al., 2011; Cunningham et al., 2013). However, its importance, function, and interactions are yet to be fully uncovered.

Microglial cells constitute the main mesoderm-derived macrophage population of the central nervous system (CNS) (Prinz and Mildner, 2011) and are distinguished from other CNS cell types by their small cell soma, as well as by the expression of specific macrophage markers (Vilhardt, 2005). Monocytes precursors generated in the yolk sac invade the early embryonic nervous parenchyma as ameboid microglial cells (Chan et al., 2007; Ginhoux et al., 2010, 2013). As development progresses, microglia within the CNS parenchyma undergo differentiation, changing from ameboid morphology into ramified cells, rather deceitfully known as resting state (Nimmerjahn et al., 2005). Ramified microglia are typically distributed throughout the adult, healthy CNS (Imamoto and Leblond, 1978; Cuadros and Navascués, 1998; Dalmau et al., 2003; Hanisch and Kettenmann, 2007). In the course of an insult microglia revert to an ameboid morphology, which usually indicates their active state (Perry et al., 1993; Hanisch and Kettenmann, 2007). Moreover, microglia are involved in several events of brain development, such as phagocytosis, neurito- and synaptogenesis, synaptic pruning, myelination, astrocyte proliferation and differentiation, and vasculogenesis (Giulian et al., 1988; Pow et al., 1989; Hamilton and Rome, 1994; Presta et al., 1995; Honda et al., 1999; Navascués et al., 2000; Streit, 2001; Rochefort et al., 2002; Marín-Teva et al., 2004; Shin et al., 2004; Checchin et al., 2006; Bessis et al., 2007; Nakanishi et al., 2007; Paolicelli et al., 2011; Kettenmann et al., 2013). Recently, microglia have also been shown to play an important role regulating neural progenitor physiology (Monje et al., 2003; Ziv et al., 2006; Sierra et al., 2010; Arnò et al., 2014; Su et al., 2014).

Here we investigate the ontogenesis, distribution and cellular interactions of microglia residing in the early postnatal SVZ, and its anterior extension, the rostral migratory stream (RMS). This region represents the major neurogenic niche in the mammalian brain that generates mostly interneurons destined for the olfactory bulb (OB) from birth to senescence (Altman, 1969; Luskin, 1993; Lois and Alvarez-Buylla, 1994). During the first two postnatal weeks a peak on proliferation is observed within the SVZ and its main progenitor cell, the radial glia (RG), undergo a process known as astrocytic transformation (Voigt, 1989; Misson et al., 1991; Freitas et al., 2012). During astrocytic transformation a set of RG transforms into astrocytes destined to populate the overlying mantle layers and/or into resident astrocytes of the SVZ/RMS pathway. Our results reveal that at this critical period microglia is already present in this germinal layer and greatly outnumber the microglia cells observed in the overlying cerebral cortex (CTX). Besides, SVZ/RMS microglia exhibit a more protracted differentiation rate compared to the regions outside this germinal zone. Importantly, during the first postnatal week SVZ/RMS microglia interact with RGs, the putative stem cells of this niche, possibly using RG processes as scaffold for its migration. Furthermore, SVZ/RMS microglia seem to engulf RGs processes, thus playing a key role in RG astrocytic transformation and possibly acting on progenitor regulation.

## Materials and methods

### Experimental animals

CX3CR1-encoding the green fluorescent protein (EGFP) mice on the C57BL/6J background were purchased from Jackson Labs (Strain Name B6.129P-CX3CR1tm1Litt/J, stock number 005582). Wild type Swiss mice, raised in our own colony, were also used. For both strains, mice at postnatal day (P) 0 up to P7 were used in our analysis. All experiments were performed in conformity with NIH (National Institute of Health, USA) guidelines for animal care and in accordance with protocols approved by both, the Animal Use Committees at the University of Rochester (UCAR-2011-021) and the Committee of Ethics on Animal Handling and Care at the Federal University of Rio de Janeiro (CEUA/DAHEICB 052; ICB/CCS—UFRJ).

#### Tissue harvesting

Heterozygous animals (CX3CR1^+^/EGFP^+^) and Swiss mice at P1 and P7 (*n* = 12 animals for each age, both strains) were deeply anesthetized by isoflurane inhalation (chamber atmosphere containing 4% isoflurane). Upon cessation of reflexes, mice were transcardially perfused with phosphate buffered saline 0.1 M (PBS, pH 7.4, Sigma Aldrich) and paraformaldehyde 4% (PFA, Sigma Aldrich, in PBS 0.1 M pH 7.4). Brains were dissected and post fixed in PFA 4% for 3–6 h at room temperature (RT). Histological sections (50–100 μm) were obtained in vibratome (Vibratome Series 3000, Vibratome Co.) and kept in PBS containing azide 0.1% (Fisher Scientific) at 4°C for immunohistochemistry analysis (see Section Immunohistochemistry).

#### BrdU administration

Short pulses of the thymidine analog BrdU (5-bromo-2′-deoxyuridine; Sigma Aldrich) were performed in order to evaluate microglia proliferation along the SVZ niche. CX3CR1-EGFP mice (P1 and P7, *n* = 6 animals for each age) received a single pulse of BrdU into the intraperitoneal cavity (i.p. injection; 150 mg Kg^−1^) and were euthanized 1 h after BrdU administration. Proliferative cells were revealed by immunohistochemistry (described below), using a primary antibody that reacts with BrdU incorporated into single stranded DNA.

### Immunohistochemistry

Histological sections were blocked for 1 h at RT in a PBS containing 0.1% Triton-X (Sigma Aldrich) solution added with 5% normal donkey serum (NDS, Vector Labs) and incubation with specific antibodies against microglial/monocyte markers (Iba1; 1:500, Wako, CD68, F4/80 and CD11c; 1:100, AbD Serotec), neuroblasts (DCX; 1:1000, Millipore) and astroglial lineage cells (GFAP; 1:250, Sigma Aldrich) was performed overnight at 4°C. Proliferative cells were revealed by using an anti-BrdU antibody (1:100, AbD Serotec). To allow labeling of nuclear DNA, before blockage, sections were treated for 1 h with HCl 1M (RT) under agitation (Tang et al., 2007). Staining was revealed by 2-hour incubation period (RT) with appropriated secondary antibodies conjugated to Cy3 or Cy5 fluorophores (1:250, Jackson ImmunoResearch). DAPI (4′,6-Diamidino-2-phenylindole, 1:1000, Sigma Aldrich) was used for nuclear counterstaining and slides were mounted with ProLong Antifade (Life Technologies). Immunolabeled brain sections were analyzed and imaged using a confocal microscope (Olympus FluoView 500) with 40x (NA 1.30) and 60x oil-immersion (NA 1.25) objective lens (Olympus). Acquired images were adjusted for brightness and contrast using FIJI/ImageJ software.

### Fluoro-gold tracer injections

Pups (P0 or P1) were anesthetized by isoflurane inhalation (chamber atmosphere containing 4% isoflurane), and under visual guidance, 100–200 nl of Fluoro-Gold (FG; hydroxystilbamidine methanesulfonate in 2% in deionized water; Fluorochrome, Englewood, CO) were injected unilaterally in the pial surface (1–0.5 mm from midline and 0.5 mm anterior to Bregma) using a glass micropipette (80–100 mm tip diameter) coupled to a pressure injector (Nanoliter 2000, WPI, Sarasota, USA). Animals were analyzed 2 or 7 days after pial injections (*n* = 6; injection site included cortical supragranular layers; animals with deep injections reaching the cortical subgranular layers or the white matter were discarded from analysis).

### Statistical analysis

Histograms are expressed as mean ± standard error (SEM). Raw data, obtained in distinct experimental approaches used in the present work, were statistically analyzed using Prism (GraphPad Software, Inc.).

## Results

### Analysis of CX3CR1-EGFP^+^ cells depicts microglia as a cellular component of the early postnatal SVZ/RMS

**Figure 1:**
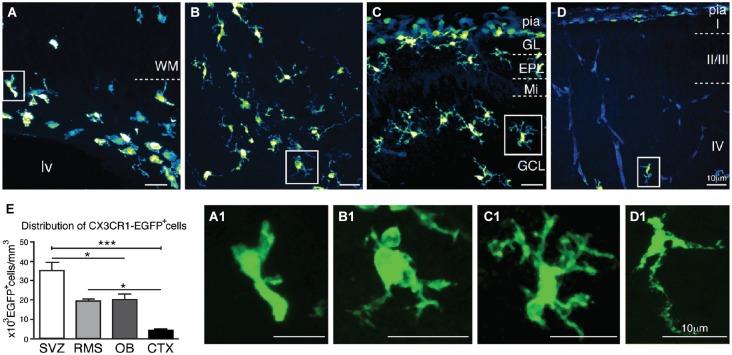
**Characterization of CX3CR1-EGFP^+^ cells morphology and distribution in newborn mice (P1)**. CX3CR1-EGFP^+^ microglia accumulate at the ventricular layers **(A)**. Interestingly, the core of RMS has a significative number of microglia cells **(B)**, as well as the distinct OB layers **(C)**. In contrast, we observe very few CX3CR1-EGFP^+^ cells in the cortical parenchyma **(D)**. As expected, CX3CR1-EGFP expressing cells exhibit immature/amoeboid morphology in all analyzed regions **(A1,B1,C1,D1). (E)** CX3CR1-EGFP^+^ cell density in newborn mice: SVZ: 35 × 10^3^ ± 4.3 × 10^3^; RMS: 19.3 × 10^3^ ± 1.3 × 10^3^; OB: 20 × 10^3^ ± 3 × 10^3^; CTX: 4.4 × 10^3^ ± 0.6 × 10^3^; cells/mm^3^; mean ± SEM; *p* < 0.05 (*) for SVZ in comparison to RMS and OB, and for RMS and OB in comparison to CTX, and *p* < 0.005 (***) for SVZ in comparison to CTX; 1way ANOVA Bonferroni’s Multiple Comparison Test]. CTX, cerebral cortex; EPL: external plexiform layer; GCL: granular cell layer; GL: glomerular layer; lv: lateral ventricle; Mi: mitral layer; OB: olfactory bulb; RMS: rostral migratory stream; SVZ: subventricular zone; WM: white matter. Scale bars: 10 μm.

Confocal microscopy analysis of brain sections obtained from newborn mice (P1) reveals that CX3CR1-EGFP^+^ cells accumulate at the ventricular layers, VZ/SVZ (Figure 1A). CX3CR1-EGFP^+^ cells are also distributed in the RMS core (Figure 1B), and within the OB layers (Figure 1C). In contrast, we observe very few CX3CR1-EGFP^+^ cells in the cortical parenchyma (Figure 1D). In common, CX3CR1-EGFP^+^ expressing cells in the SVZ, RMS, OB and CTX display immature/amoeboid morphology (Figures 1A1–D1), regardless of the significative difference on cell density between these regions [SVZ: 35 × 10^3^ ± 4.3 × 10^3^; RMS: 19.3 × 10^3^ ± 1.3 × 10^3^; OB: 20 × 10^3^ ± 3 × 10^3^; CTX: 4.4 × 10^3^ ± 0.6 × 10^3^; CX3CR1-EGFP^+^ cells/mm^3^; mean ± SEM; *p* < 0.05 for SVZ in comparison to RMS and OB, and for RMS and OB in comparison to CTX, and *p* < 0.005 for SVZ in comparison to CTX; 1way ANOVA Bonferroni’s Multiple Comparison Test] (Figure 1E).

**Figure 2:**
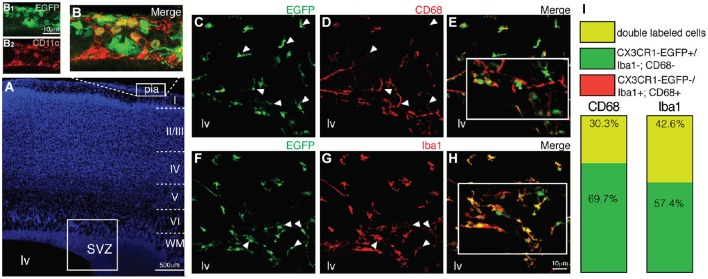
**Antigenic heterogeneity of CX3CR1-EGFP^+^ cells morphology in early postnatal SVZ. (A)** DAPI counterstaining unveils the cytoarchitecture of cortical parenchyma and ventricular region. Dendritic cells (CD11c^+^, red) are restricted to the pial surface and some of them co-express CX3CR1-EGFP **(B,B1,B2)**. Immunolabeling of brain sections obtained from CX3CR1-EGFP mice with CD68 (**C–E**, red) and Iba1 (**F–H**, red) demonstrate that some of EGFP^+^ cells (green) are not co-labeled by these common used microglia markers (white arrowheads). **(I)** The percentage of CX3CR1-EGFP^+^/CD68^+^ cells is 30.3% of the total of microglia observed and of CX3CR1-EGFP^+^/Iba1^+^ cells is 42.6%. SVZ: subventricular zone. Scale bars: 100 μm **(A)** and 10 μm (**B1,B2,C–H**).

We next asked if the CX3CR1-EGFP^+^ cells observed within the SVZ/RMS niche, OB and in the cortical parenchyma correspond solely to microglial cells, as the fractalkine receptor is also expressed by monocytes, subsets of natural killers and dendritic cells (Jung et al., 2000). Since the dendritic cell antigen CD11c was detected in a transgenic mice strain in postnatal SVZ cells that were also immunoreactive for microglial markers (Bulloch et al., 2008), we analyzed by immunohistochemistry if CX3CR1-EGFP^+^ cells were co-labeled by CD11c. Notably, the majority of CX3CR1-EGFP expressing cells present in the SVZ/RMS, OB and cortical parenchyma correspond to microglia, as only a few cells restricted to the pial surface, are co-labeled by dendritic cell marker CD11c (Figures 2A,B,B1,B2).

Remarkably, analysis of brain sections obtained from CX3CR1-EGFP mice demonstrates that part of CX3CR1-EGFP^+^ cells are not co-labeled by CD68 (Figures 2C–E). Of the total of microglial cells observed within the SVZ, CX3CR1-EGFP^+^/CD68^+^ cells corresponded to 30.3% and CX3CR1-EGFP^+^/CD68^−^ cells corresponded to 69.7% (Figure 2I). No CX3CR1-EGFP^−^/CD68^+^ cells were observed. Similarly, immunostaining for Iba1 revealed that 42.6% of the microglia in the SVZ are CX3CR1-EGFP^+^/Iba1^+^ and 57.4% CX3CR1-EGFP^+^/Iba1^−^ (Figures 2F–I), indicating that the SVZ microglia are a heterogeneous population (Olah et al., 2011).

**Figure 3:**
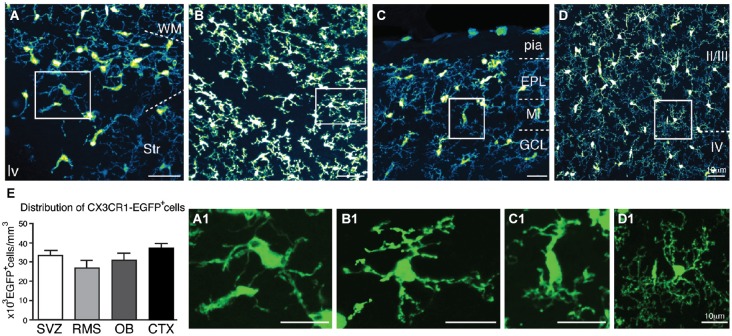
**Characterization of CX3CR1-EGFP^+^ microglia morphology and density in neonatal mice**. In P7 mice, microglia present in the SVZ retains immature morphology **(A,A1)**, differing from EGFP^+^ cells present in the adjacent areas (WM and Str). In the RMS, CX3CR1-EGFP^+^ cells also display immature/migratory morphology **(B,B1)**. Similar morphology is shown by CX3CR1-EGFP^+^ cells in the OB **(C,C1)**. Within CTX layers, ramified CX3CR1-EGFP^+^ microglia are homogenously distributed **(D,D1). (E)** At this age, no significative differences on CX3CR1-EGFP^+^ cell number are observed in the analyzed regions [SVZ: 33 × 10^3^ ± 3 × 10^3^; RMS: 26.9 × 10^3^ ± 3.7 × 10^3^; OB: 30 × 10^3^ ± 3.7 × 10^3^; CTX: 36 × 10^3^ ± 2.6 × 10^3^; cells/mm^3^; mean ± SEM; *p* > 0.05, 1way ANOVA Bonferroni’s Multiple Comparison Test]. EPL: external plexiform layer; GCL: granular cell layer; lv: lateral ventricle; Mi: mitral layer; WM: white matter. Scale bars: 10 μm.

**Figure 4:**
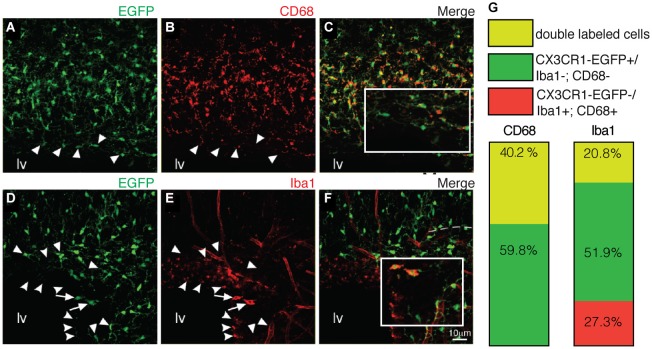
**Macrophage markers cannot depict the entire SVZ microglial population. (A–C)** Similar to newborn mice, some of EGFP^+^ cells (green) are not co-labeled by CD68 (red) (indicated by arrowheads). CX3CR1-EGFP^+^/CD68^+^ cells correspond to 40.2% of the microglia in the SVZ **(G)**. Immunostaining with Iba1 (**D–F**, red) reveals that CX3CR1-EGFP^+^/Iba1^+^ cells (arrows) correspond to 20.8%. and CX3CR1-EGFP^+^/Iba1^−^ cells (arrowheads) represent 51.9% of microglia **(G)**. Notably, some cells are solely labeled by Iba1, corresponding to 27.3% of the SVZ microglia (round arrowheads). lv: lateral ventricle. Scale bars: 10 μm.

At later stages (P7), CX3CR1-EGFP expressing microglia present in the SVZ retains their immature morphology (Figures 3A,A1). Despite the dense population of ramified microglia outside its borders, CX3CR1-EGFP^+^ cells along the RMS also display immature/migratory morphology (Figures 3B,B1), similar to the microglial cells distributed within the OB layers (Figures 3C,C1). In contrast, we observe ramified CX3CR1-EGFP^+^ microglia spanning all the cortical layers (Figures 3D,D1), and at this age, no significative differences on CX3CR1-EGFP^+^ cell number are observed when comparing all analyzed regions [SVZ: 33 × 10^3^ ± 3 × 10^3^; RMS: 26.9 × 10^3^ ± 3.7 × 10^3^; OB: 30 × 10^3^ ± 3.7 × 10^3^; CTX: 36 × 10^3^ ± 2.6 × 10^3^; CX3CR1-EGFP^+^ cells/mm^3^; mean ± SEM; *p* > 0.05, 1way ANOVA Bonferroni’s Multiple Comparison Test] (Figure 3E). Likewise observed in newborn mice, CX3CR1-EGFP^+^ cells in the SVZ/RMS, regarding their immunoreactivity, remain a quite heterogeneous population at P7. Our results show that CX3CR1-EGFP^+^/CD68^+^ cells corresponded to 40.2% and CX3CR1-EGFP^+^/CD68^−^ cells represent 69.7% of microglia present in the SVZ (Figures 4A–C,G). Analysis of Iba1 immunoreactivity shows that CX3CR1-EGFP^+^/Iba1^+^ cells correspond to 20.8% of the microglia, whereas CX3CR1-EGFP^−^/Iba1^+^ cells correspond to 27.3%. However, the majority of microglia in the SVZ is CX3CR1-EGFP^+^/Iba1^−^ cells, corresponding to 51.9% (Figures 4D–G).

### Microglial cells residing in the SVZ niche are not proliferative during early neonatal stages

**Figure 5:**
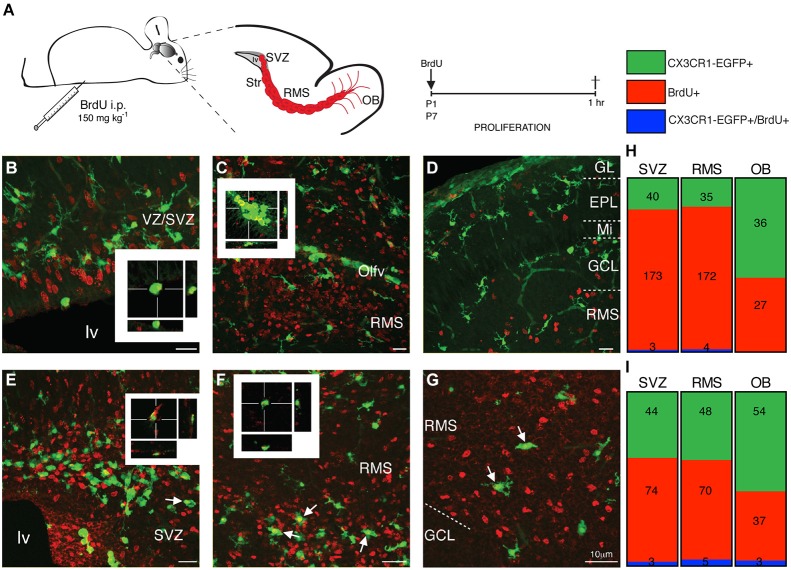
**Microglia residing in neonatal the SVZ, RMS and OB are quiescent cells. (A)** Schematic representation of brain parasagittal section obtained from neonatal mice that received a single, short pulse of BrdU (i.p. injections of BrdU; 150 mg kg^−1^). Sections containing the SVZ, RMS and OB were processed for BrdU immunostaining. **(B)** Dividing (BrdU^+^ cells, red) are observed in the ventricular layers of P1 mice, but majority of SVZ microglia do not incorporate BrdU and only few cells are EGFP^+^ (green)/BrdU^+^, as depicted by optical sectioning. In the RMS of P1 mice, CX3CR1-EGFP^+^/BrdU^+^ cells are restricted into the olfactory ventricle **(C)**, while in the OB, we do not observe any CX3CR1-EGFP^+^/BrdU^+^ cell at this stage **(D)**. At P7, CX3CR1-EGFP^+^ cells intermingle BrdU^+^ cells, but few proliferative microglia are observed in the SVZ (**E**, arrows) and in the borders of RMS (**F**, arrows). Within OB layers, few CX3CR1-EGFP^+^ cells do not incorporate BrdU (**G**, arrows). Quantification is shown, in absolute numbers, in **H** and **I**. EPL: external plexiform layer; GCL: granular cell layer; GL: glomerular layer; lv: lateral ventricle; Mi: mitral layer; OB: olfactory bulb; Olfv: olfactory ventricle; RMS: rostral migratory stream; SVZ: subventricular zone; WM: white matter; VZ: ventricular zone. Scale bars: 10 μm.

Once inside the CNS, microglia precursors spread within the neural tissue, a process that includes cell proliferation and/or migration. In order to determine the spreading dynamics of microglial cells in the SVZ niche, we accessed the proliferation status of CX3CR1- EGFP^+^ cells in the SVZ, RMS and OB during the first postnatal week. After a short pulse of BrdU (1 h before euthanasia), scarce CX3CR1-EGFP^+^/BrdU^+^ cells are observed in the lateral (lv) and olfactory ventricles (Olfv) of P1 mice (Figures 5B,C, respectively, and Figure 5H). Within the OB layers, only CX3CR1-EGFP^+^/BrdU^−^ are observed, indicating that the majority of microglial cells in the neonatal SVZ niche is quiescent (Figures 5D,H). At P7, we observe few CX3CR1-EGFP^+^/BrdU^+^ cells in the ventricular region (Figures 5E,I). Along the RMS, dividing microglia are detected in its borders, as well as some BrdU fragments are engulfed by microglia (Figures 5F,I). Remarkably, in the OB BrdU^+^ cells distributed along the distinct layers are sparsely contacted by CX3CR1-EGFP^+^ microglia and some CX3CR1-EGFP^+^/BrdU^+^ are observed (Figures 5G,I).

### Microglia cellular interactions within the neonatal SVZ/RMS niche

**Figure 6:**
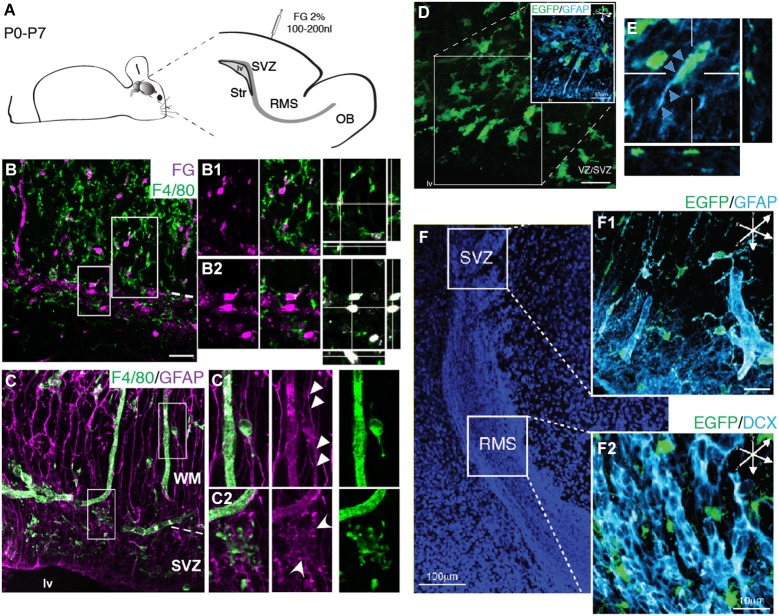
**Microglia cellular interactions within the SVZ niche. (A)** Schematic representation of brain parasagittal section obtained from neonatal Swiss mice injected with FG (2%) in the pial surface. **(B)** Histological sections of injected mice and immunolabeled for F4/80 antigen reveal the overlapping of FG^+^ cell soma (magenta) and F4/80^+^ cells (green) in the SVZ and in the WM. RGs present in both the SVZ and overlaying WM are depicted in higher magnification in **(B1)** and **(B2)**, respectively. Optical sectioning shows that FG^+^ cells are engulfed by F4/80^+^ microglia. **(C)** Microglia (F4/80^+^ cells, green) appose to RG processes (GFAP, magenta) that span the cortical parenchyma. **(C1)** Microglia displaying migratory morphology appose to RG processes spanning the WM (arrowheads). **(C2)** Within the SVZ, microglia displaying immature morphology are closely associated to GFAP^+^ astroglial cells (round arrowheads). **(D)** In transgenic mice, CX3CR1-EGFP^+^ cells (green) also associate to RG processes (GFAP, cyan), displaying migratory morphology, thus using astroglial processes as scaffold to invade the cortical parenchyma. **(E)** Orthogonal view of CX3CR1-EGFP^+^ cells along GFAP^+^ processes (arrowheads). **(F)**. SVZ and RMS delimit the cell dense region counterstained with DAPI. Within the SVZ, CX3CR1-EGFP^+^ cells intermingle GFAP^+^ cells **(F1)**, whereas in the RMS, microglia cells interleave neuroblasts chains (**F2**, DCX^+^ cells, cyan).

We next sought to determine the microglial interactions with the typical cell types observed within the SVZ niche, namely the astroglial stem cell lineage (RGs and stem cell-like astrocytes/type B cells) and neuroblasts. Since previous studies show that tracer injections at the pial surface labels exclusively RG within the SZV (Freitas et al., 2012), we took advantage of the fact that at birth many RGs still maintain a long process touching the pial surface (Misson et al., 1991; Alves et al., 2002) to label this cell population. The neuroanatomical tracer Fluoro-Gold was injected in the pial surface of newborn mice (P0) and we followed labeled RGs up to the first postnatal week (P7). Immunohistochemistry analysis of brain sections obtained from injected animals reveals the transcellular labeling of microglia (F4/80^+^ cells) neighboring labeled RG (Figures 6B,B1,B2). This is suggestive of a very intimate contact of microglia with RG, although we could not distinguish if this transcellular labeling was due to whole engulfment of RG by microglia, or partial phagocytosis of RG processes. Immunolabeling of RGs and astrocytes with GFAP antibody reveal a close apposition of microglia to astroglial processes (Figure 6C). Some microglia display a migratory morphology (Figures 6C1,D,E), indicating that microglia use radial processes to migrate within the cortical parenchyma. Remarkably, we also observe microglial cells enfolding GFAP^+^ processes in the SVZ/WM border, where GFAP^+^ cells accumulate during their putative astroglial transformation (Figure 6C2). Interestingly, along the SVZ/RMS of neonatal mice (P7) microglia are conspicuously distributed, most often intermingling with the astrocyte compartment and outside of the chains of migratory neuroblasts (Figures 6F,F1,F2).

## Discussion

Here we demonstrate that microglia present within the early postnatal SVZ represents a copious population, which outnumber cortical microglia population during neonatal stages (Figure 1E). Our observations also show that SVZ microglia exhibit a remarkable antigenic plasticity (Figures 2, 4) and quiescence (Figure 5), confirming and extending the concept of microglia regional heterogeneity (Carson et al., 2007; Olah et al., 2011). Furthermore, our analysis reveal that microglia is intimately associated to the astroglial compartment within the SVZ, since dye transfer between RG cells and microglial cells were observed, possibly a result of phagocytosis, and a spatial overlap with GFAP positive cells (Figure 6). This interaction could represent a direct microglia control over late cortical progenitors of the outer SVZ (Franco et al., 2012) or progenitors for interneurons of the OB layers (Merkle et al., 2007; Ventura and Goldman, 2007). Alternatively, suggest that microglia is involved in the astrocytic transformation of a subset of RG cells in the early postnatal SVZ/RMS as suggested in earlier publications (Schmechel and Rakic, 1979; Voigt, 1989; Misson et al., 1991; Alves et al., 2002; Freitas et al., 2012).

For several years, the presence of microglia within the early postnatal SVZ/RMS was either neglected (Dalmau et al., 2003), or undetected (Peretto et al., 2005). Only recently has microglia within the early SVZ been investigated (Shigemoto-Mogami et al., 2014). The previous underestimation of microglia within germinative layers, and specifically in the postnatal SVZ, may be due to the great phenotypic plasticity of microglia cells (Saijo and Glass, 2011), making detection by usual phenotypic markers unreliable. We have circumvented this limitation by using a transgenic animal in which the reporter gene encoding the green fluorescent protein (EGFP) was introduced in the locus of the constitutively expressed fractalkine CX3CR1 receptor (Jung et al., 2000), yielding a stable marker for this population. This results in a golgi-like cell labeling with EGFP, throughout the developing brain parenchyma, displaying several chracteristics of microglia. CX3CR1-EGFP^+^ microglia in the SVZ were neither immunoreactive for neural nor blood vessel markers (data not shown), and also unlabeled by a dendritic cell marker CD11c. Interestingly and rather unexpected, common used markers for microglia only partially co-localize with CX3CR1-EGFP^+^ cells. Since most of phenotypic markers used to reveal macrophages are membrane molecules related to cell-cell or cell-extracellular milieu interactions (Ling et al., 1991; Milligan et al., 1991; Chen et al., 2002; Gomez Perdiguero et al., 2013), this variability of antigen expression may reflect the influence of discrete signals present within this neurogenic niche, which may instruct and control identity and specialization of microglia.

The most striking morphological feature observed for microglia within the neonatal SVZ is their characteristic activated profile (Figures 1, 3A), exhibiting ameboid morphology with few thick and short branches, also typical of immature microglia (Perry et al., 1993; Hanisch and Kettenmann, 2007). At P7, when cortical microglia already display a ramified “resting” morphology (Lima et al., 2001; Dalmau et al., 2003), SVZ microglia still retains the immature/activated profile. This may be a common feature for microglia resident of germinative layers, since the same reactive profile has been described for the embryonic cerebral cortical VZ/SVZ (Cunningham et al., 2013) and adult SVZ (Goings et al., 2006). Another outstanding difference of SVZ microglia is their relative quiescence (Figure 5), in contrast to actively proliferating microglia distributed throughout the cortical parenchyma during the first postnatal week (Mallat et al., 1997; Alliot et al., 1999; Dalmau et al., 2003). These regional differences could result from signals emanating from a progenitor enriched environment that has been shown to instruct resident microglia (Mosher et al., 2012; Linnartz and Neumann, 2013). It remains to be determined if this microglia behavior is dynamically controlled or represents an irreversible phenotype. It is interesting to note that microglia harvested from the adult SVZ, behave differently in culture, even after many *in vitro* passages (Walton et al., 2006), suggesting some stable and environment independent features for this microglial population.

A straightforward mechanism for any putative function for microglia over SVZ/RMS progenitors could lie on their intrinsic phagocytic activity. Phagocytosis of neural progenitors has been shown to occur in the subgranular layer of the dentate gyrus (Sierra et al., 2010) and in the embryonic telencephalic ventricular zone (Cunningham et al., 2013). To test this hypothesis we have retrogradely labeled RG cells present in the SVZ by injecting the fluorescent tracer Fluoro-Gold at the pial surface. Previous results have shown that 2 days after pial injection of anatomical tracers only RG are labeled within the SVZ (Freitas et al., 2012). Interestingly, at 7 days post injection, we find the labeling of microglia. This can be explained as reminiscent of transcellular transfer of dyes to microglia by phagocytosis of retrogradely labeled cells, as observed in other systems (Thanos et al., 2000). Together this data suggests that microglia is actively phagocytizing RGs. However, alternatively this transcellular labeling may be due to gap junctional communication (Freitas et al., 2012) or partial phagocytosis of RG processes in a manner analogous to microglia stripping of neuronal synapses and processes described previously (Kettenmann et al., 2013). Although further investigation may be necessary to distinguish between these possibilities, the transcellular dye transfer and overlap of distribution with the astroglial compartment, as shown by double labeling with GFAP (Figure 6C2), indicates a consistent interaction between microglia and RGs. Nevertheless, we cannot rule out the hypothesis that microglia may also be phagocytizing neuroblasts en route to the OB layers, as shown to occur at the hippocampus dentate gyrus (Sierra et al., 2010).

Given their rapid response to diffusible signals and cell-cell interactions, microglia may represent a pivotal player to integrate short and long-range environmental cues within the germinal layers (Su et al., 2014). It has been documented that microglia can respond to neurotransmitters (Fontainhas et al., 2011); trophic factors (Ryu et al., 2012); peripheral cytokines and chemokines (Butovsky et al., 2006); humoral signaling from disease (Li and Graeber, 2012; Tsuda et al., 2013; Yu and Ye, 2014; Hu et al., 2015), membrane glycocalyx (Linnartz and Neumann, 2013) and progenitor secreted proteins (Mosher et al., 2012). On the executive side, microglia could exert their influence not only by its phagocytic activity, engulfing whole cells, processes, or stripping membranes (Kettenmann et al., 2013), but through the release of cytokines and trophic factors and (Nakajima et al., 2007; Cacci et al., 2008; Liao et al., 2008; Ueno et al., 2013). It is still unclear what specific roles microglia play over the generation (Shigemoto-Mogami et al., 2014), migration (Aarum et al., 2003) and addition of new neurons to the OB (Lazarini et al., 2012) and in response to insult (Goings et al., 2006). However, given the possible action of the selective phagocytosis of precursors and progeny (Sierra et al., 2010; Cunningham et al., 2013) microglia activity could contribute to the mismatch observed between the very restricted generative potential of SVZ neural progenitors *in situ* (Luskin, 1993; Lim and Alvarez-Buylla, 2014) and its wider capabilities when challenged *in vivo* or *in vitro* (Sequerra et al., 2010, 2013).

## Conflict of interest statement

The authors declare that the research was conducted in the absence of any commercial or financial relationships that could be construed as a potential conflict of interest.

## References

[B1] Aarum J. Sandberg K. Haeberlein S. L. Persson M. A. (2003). Migration and differentiation of neural precursor cells can be directed by microglia. Proc. Natl. Acad. Sci. U S A 100, 15983–15988. 10.1073/pnas.2237050100 14668448 PMC307679

[B2] Alliot F. Godin I. Pessac B. (1999). Microglia derive from progenitors, originating from the yolk sac and which proliferate in the brain. Brain Res. Dev. Brain Res. 117, 145–152. 10.1016/s0165-3806(99)00113-3 10567732

[B3] Altman J. (1969). Autoradiographic and histological studies of postnatal neurogenesis. IV. Cell proliferation and migration in the anterior forebrain, with special reference to persisting neurogenesis in the olfactory bulb. J. Comp. Neurol. 137, 433–457. 10.1002/cne.901370404 5361244

[B4] Alves J. A. Barone P. Engelender S. Fróes M. M. Menezes J. R. L. (2002). Initial stages of radial glia astrocytic transformation in the early postnatal anterior subventricular zone. J. Neurobiol. 52, 251–265. 10.1002/neu.10087 12210108

[B5] Arnò B. Grassivaro F. Rossi C. Bergamaschi A. Castiglioni V. Furlan R.. (2014). Neural progenitor cells orchestrate microglia migration and positioning into the developing cortex. Nat. Commun. 5:5611. 10.1038/ncomms6611 25425146

[B6] Bessis A. Béchade C. Bernard D. Roumier A. (2007). Microglial control of neuronal death and synaptic properties. Glia 55, 233–238. 10.1002/glia.20459 17106878

[B7] Bulloch K. Miller M. M. Gal-Toth J. Milner T. A. Gottfried-Blackmore A. Waters E. M.. (2008). CD11c/EYFP transgene illuminates a discrete network of dendritic cells within the embryonic, neonatal, adult and injured mouse brain. J. Comp. Neurol. 508, 687–710. 10.1002/cne.21668 18386786

[B8] Butovsky O. Ziv Y. Schwartz A. Landa G. Talpalar A. E. Pluchino S.. (2006). Microglia activated by IL-4 or IFN-gamma differentially induce neurogenesis and oligodendrogenesis from adult stem/progenitor cells. Mol. Cell. Neurosci. 31, 149–160. 10.1016/j.mcn.2005.10.006 16297637

[B9] Cacci E. Ajmone-Cat M. A. Anelli T. Biagioni S. Minghetti L. (2008). In vitro neuronal and glial differentiation from embryonic or adult neural precursor cells are differently affected by chronic or acute activation of microglia. Glia 56, 412–425. 10.1002/glia.20616 18186084

[B10] Carson M. J. Bilousova T. V. Puntambekar S. S. Melchior B. Doose J. M. Ethell I. M. (2007). A rose by any other name? The potential consequences of microglial heterogeneity during CNS health and disease. Neurotherapeutics 4, 571–579. 10.1016/j.nurt.2007.07.002 17920538 PMC2637868

[B11] Chan W. Y. Kohsaka S. Razaie P. (2007). The origin and cell lineage of microglia—new concepts. Brain Res. Rev. 53, 344–354. 10.1016/j.brainresrev.2006.11.002 17188751

[B12] Checchin D. Sennlaub F. Levavasseur E. Leduc M. Chemtob S. (2006). Potential role of microglia in retinal blood vessel formation. Invest. Ophthalmol. Vis. Sci. 47, 3595–3602. 10.1167/iovs.05-1522 16877434

[B84] Chen L. Yang P. Kijlstra A. (2002). Distribution, markers, and functions of retinal microglia. Ocul. Immunol. Inflamm. 10, 27–39. 10.1076/ocii.10.1.27.10328 12461701

[B13] Cuadros M. A. Navascués J. (1998). The origin and differentiation of microglial cells during development. Prog. Neurobiol. 56, 173–189. 10.1016/s0301-0082(98)00035-5 9760700

[B14] Cunningham C. L. Martínez-Cerdeño V. Noctor S. C. (2013). Microglia regulate the number of neural precursor cells in the developing cerebral cortex. J. Neurosci. 33, 4216–4233. 10.1523/jneurosci.3441-12.2013 23467340 PMC3711552

[B15] Dalmau I. Vela J. M. González B. Finsen B. Castellano B. (2003). Dynamics of microglia in the developing rat brain. J. Comp. Neurol. 458, 144–157. 10.1002/cne.10572 12596255

[B88] Fontainhas A. M. Wang M. Liang K. J. Chen S. Mettu P. Damani M.. (2011). Microglial morphology and dynamic behavior is regulated by ionotropic glutamatergic and GABAergic neurotransmission. PLoS One 6:e15973. 10.1371/journal.pone.0015973 21283568 PMC3026789

[B16] Franco S. J. Gil-Sanz C. Martinez-Garay I. Espinosa A. Harkins-Perry S. R. Ramos C.. (2012). Fate-restricted neural progenitors in the mammalian cerebral cortex. Science 337, 746–749. 10.1126/science.1223616 22879516 PMC4287277

[B17] Franco S. J. Müller U. (2013). Shaping our minds: stem and progenitor cell diversity in the mammalian neocortex. Neuron 77, 19–34. 10.1016/j.neuron.2012.12.022 23312513 PMC3557841

[B18] Freitas A. S. Xavier A. L. Furtado C. M. Hedin-Pereira C. Fróes M. M. Menezes J. R. L. (2012). Dye coupling and connexin expression by cortical radial glia in the early postnatal subventricular zone. Dev. Neurobiol. 72, 1482–1497. 10.1002/dneu.22005 22234946

[B19] Ginhoux F. Greter M. Leboeuf M. Nandi S. See P. Gokhan S.. (2010). Fate mapping analysis reveals that adult microglia derive from primitive macrophages. Science 330, 841–845. 10.1126/science.1194637 20966214 PMC3719181

[B20] Ginhoux F. Lim S. Hoeffel G. Low D. Huber T. (2013). Origin and differentiation of microglia. Front. Cell. Neurosci. 7:45. 10.3389/fncel.2013.00045 23616747 PMC3627983

[B21] Giulian D. Young D. G. Woodward J. Brown D. C. Lachman L. B. (1988). Interleukin-1 is an astroglial growth factor in the developing brain. J. Neurosci. 8, 709–714. 3257519 10.1523/JNEUROSCI.08-02-00709.1988 PMC6569312

[B85] Goings G. E. Kozlowski D. A. Szele F. G. (2006). Differential activation of microglia in neurogenic versus non-neurogenic regions of the forebrain. Glia 54, 329–342. 10.1002/glia.20381 16862532

[B22] Gomez Perdiguero E. Schulz C. Geissmann F. (2013). Development and homeostasis of “resident” myeloid cells: the case of the microglia. Glia 61, 112–120. 10.1002/glia.22393 22847963

[B23] Götz M. Huttner W. B. (2005). The cell biology of neurogenesis. Nat. Rev. Mol. Cell Biol. 6, 777–788. 10.1038/nrm1739 16314867

[B24] Hamilton S. P. Rome L. H. (1994). Stimulation of in vitro myelin synthesis by microglia. Glia 11, 326–335. 10.1002/glia.440110405 7960036

[B25] Hanisch U. K. Kettenmann H. (2007). Microglia: active sensor and versatile effector cells in the normal and pathologic brain. Nat. Neurosci. 10, 1387–1394. 10.1038/nn1997 17965659

[B26] Honda S. Nakajima K. Nakamura Y. Imai Y. Kohsaka S. (1999). Rat primary cultured microglia express glial cell line-derived neurotrophic factor receptors. Neurosci. Lett. 275, 203–206. 10.1016/s0304-3940(99)00769-7 10580710

[B27] Hu X. Leak R. K. Shi Y. Suenaga J. Gao Y. Zheng P.. (2015). Microglial and macrophage polarization-new prospects for brain repair. Nat. Rev. Neurol. 11, 56–64. 10.1038/nrneurol.2014.207 25385337 PMC4395497

[B28] Imamoto K. Leblond C. P. (1978). Radioautographic investigation of gliogenesis in the corpus callosum of young rats. II. Origin of microglial cells. J. Comp. Neurol. 180, 139–163. 10.1002/cne.901800109 649786

[B29] Jones D. L. Wagers A. J. (2008). No place like home: anatomy and function of the stem cell niche. Nat. Rev. Mol. Cell Biol. 9, 11–21. 10.1038/nrm2319 18097443

[B30] Jung S. Aliberti J. Graemmel P. Sunshine M. J. Kreutzberg G. W. Sher A.. (2000). Analysis of fractalkine receptor CX(3)CR1 function by targeted deletion and green fluorescent protein reporter gene insertion. Mol. Cell. Biol. 20, 4106–4114. 10.1128/mcb.20.11.4106-4114.2000 10805752 PMC85780

[B31] Kettenmann H. Kirchhoff F. Verkhratsky A. (2013). Microglia: new roles for the synaptic stripper. Neuron 77, 10–18. 10.1016/j.neuron.2012.12.023 23312512

[B32] Lazarini F. Gabellec M. M. Torquet N. Lledo P. M. (2012). Early activation of microglia triggers long-lasting impairment of adult neurogenesis in the olfactory bulb. J. Neurosci. 32, 3652–3664. 10.1523/jneurosci.6394-11.2012 22423088 PMC6703455

[B33] Li W. Graeber M. B. (2012). The molecular profile of microglia under the influence of glioma. Neuro Oncol. 14, 958–978. 10.1093/neuonc/nos116 22573310 PMC3408253

[B34] Liao H. Huang W. Niu R. Sun L. Zhang L. (2008). Cross-talk between the epidermal growth factor-like repeats/fibronectin 6-8 repeats domains of Tenascin-R and microglia modulates neural stem/progenitor cell proliferation and differentiation. J. Neurosci. Res. 86, 27–34. 10.1002/jnr.21454 17803220

[B35] Lim D. A. Alvarez-Buylla A. (2014). Adult neural stem cells stake their ground. Trends Neurosci. 37, 563–571. 10.1016/j.tins.2014.08.006 25223700 PMC4203324

[B36] Lima F. R. Gervais A. Colin C. Izembart M. Neto V. M. Mallat M. (2001). Regulation of microglial development: a novel role for thyroid hormone. J. Neurosci. 21, 2028–2038. 11245686 10.1523/JNEUROSCI.21-06-02028.2001 PMC6762591

[B37] Ling E. A. Kaur C. Wong W. C. (1991). Expression of major histocompatibility complex and leukocyte common antigens in amoeboid microglia in postnatal rats. J. Anat. 177, 117–126. 1769886 PMC1260419

[B87] Linnartz B. Neumann H. (2013). Microglial activatory (immunoreceptor tyrosine-based activation motif)- and inhibitory (immunoreceptor tyrosine-based inhibition motif)-signaling receptors for recognition of the neuronal glycocalyx. Glia 61, 37–46. 10.1002/glia.22359 22615186

[B38] Lois C. Alvarez-Buylla A. (1994). Long-distance neuronal migration in the adult mammalian brain. Science 264, 1145–1148. 10.1126/science.8178174 8178174

[B39] Luskin M. B. (1993). Restricted proliferation and migration of postnatally generated neurons derived from the forebrain subventricular zone. Neuron 11, 173–189. 10.1016/0896-6273(93)90281-u 8338665

[B86] Mallat M. Calvo C. F. Dobbertin A. (1997). Migration and proliferation of mononuclear phagocytes in the central nervous system. Adv. Exp. Med. Biol. 429, 99–108. 10.1007/978-1-4757-9551-6_7 9413568

[B40] Marín-Teva J. L. Dusart I. Colin C. Gervais A. van Rooijen N. Mallat M. (2004). Microglia promote the death of developing Purkinje cells. Neuron 41, 535–547. 10.1016/s0896-6273(04)00069-8 14980203

[B82] Mercier F. Kitasako J. T. Hatton G. I. (2002). Anatomy of the brain neurogenic zones revisited: fractones and the fibroblast/macrophage network. J. Comp. Neurol. 451, 170–188. 10.1002/cne.10342 12209835

[B41] Merkle F. T. Mirzadeh Z. Alvarez-Buylla A. (2007). Mosaic organization of neural stem cells in the adult brain. Science 317, 381–384. 10.1126/science.1144914 17615304

[B42] Miller F. D. Gauthier-Fisher A. (2009). Home at last: neural stem cell niches defined. Cell Stem Cell 4, 507–510. 10.1016/j.stem.2009.05.008 19497279

[B43] Milligan C. E. Cunningham T. J. Levitt P. (1991). Differential immunochemical markers reveal the normal distribution of brain macrophages and microglia in the developing rat brain. J. Comp. Neurol. 314, 125–135. 10.1002/cne.903140112 1797868

[B44] Misson J. P. Austin C. P. Takahashi T. Cepko C. L. Caviness V. S. Jr. (1991). The alignment of migrating neural cells in relation to the murine neopallial radial glial fiber system. Cereb. Cortex 1, 221–229. 10.1093/cercor/1.3.221 1668365

[B45] Monje M. L. Toda H. Palmer T. D. (2003). Inflammatory blockade restores adult hippocampal neurogenesis. Science 302, 1760–1765. 10.1126/science.1088417 14615545

[B46] Mosher K. I. Andres R. H. Fukuhara T. Bieri G. Hasegawa-Moriyama M. He Y.. (2012). Neural progenitor cells regulate microglia functions and activity. Nat. Neurosci. 15, 1485–1487. 10.1038/nn.3233 23086334 PMC3495979

[B47] Nakajima K. Tohyama Y. Maeda S. Kohsaka S. Kurihara T. (2007). Neuronal regulation by which microglia enhance the production of neurotrophic factors for GABAergic, catecholaminergic and cholinergic neurons. Neurochem. Int. 50, 807–820. 10.1016/j.neuint.2007.02.006 17459525

[B48] Nakanishi M. Niidome T. Matsuda S. Akaike A. Kihara T. Sugimoto H. (2007). Microglia-derived interleukin-6 and leukaemia inhibitory factor promote astrocytic differentiation of neural stem/progenitor cells. Eur. J. Neurosci. 25, 649–658. 10.1111/j.1460-9568.2007.05309.x 17328769

[B49] Navascués J. Calvente R. Marín-Teva J. L. Cuadros M. A. (2000). Entry, dispersion and differentiation of microglia in the developing central nervous system. An. Acad. Bras. Cienc. 72, 91–102. 10.1590/s0001-37652000000100013 10932110

[B50] Nimmerjahn A. Kirchhoff F. Helmchen F. (2005). Resting microglial cells are highly dynamic surveillants of brain parenchyma in vivo. Science 308, 1314–1318. 10.1126/science.1110647 15831717

[B51] Olah M. Biber K. Vinet J. Boddeke H. W. (2011). Microglia phenotype diversity. CNS Neurol. Disord. Drug Targets 10, 108–118. 10.2174/187152711794488575 21143141

[B52] Paolicelli R. C. Bolasco G. Pagani F. Maggi L. Scianni M. Panzanelli P.. (2011). Synaptic pruning by microglia is necessary for normal brain development. Science 333, 1456–1458. 10.1126/science.1202529 21778362

[B53] Pathania M. Yan L. D. Bordey A. (2010). A symphony of signals conducts early and late stages of adult neurogenesis. Neuropharmacology 58, 865–876. 10.1016/j.neuropharm.2010.01.010 20097213 PMC2850602

[B54] Peretto P. Giachino C. Aimar P. Fasolo A. Bonfanti L. (2005). Chain formation and glial tube assembly in the shift from neonatal to adult subventricular zone of the rodent forebrain. J. Comp. Neurol. 487, 407–427. 10.1002/cne.20576 15906315

[B55] Perry V. H. Anderson P. B. Gordon S. (1993). Macrophages and inflammation in the central nervous system. Trends Neurosci. 16, 268–273. 10.1016/0166-2236(93)90180-t 7689770

[B56] Pow D. V. Perry V. H. Morris J. F. Gordon S. (1989). Microglia in the neurohypophysis associate with and endocytose terminal portions of neurosecretory neurons. Neuroscience 33, 567–578. 10.1016/0306-4522(89)90409-0 2636710

[B57] Presta M. Urbinati C. Dell’era P. Lauro G. M. Sogos V. Balaci L.. (1995). Expression of basic fibroblast growth factor and its receptors in human fetal microglia cells. Int. J. Dev. Neurosci. 13, 29–39. 10.1016/0736-5748(94)00065-b 7793308

[B58] Prinz M. Mildner A. (2011). Microglia in the CNS: immigrants from another world. Glia 59, 177–187. 10.1002/glia.21104 21125659

[B59] Rochefort N. Quenech’du N. Watroba L. Mallat M. Giaume C. Milleret C. (2002). Microglia and astrocytes may participate in the shaping of visual callosal projections during postnatal development. J. Physiol. Paris. 96, 183–192. 10.1016/s0928-4257(02)00005-0 12445895

[B60] Ryu K. Y. Cho G. S. Piao H. Z. Kim W. K. (2012). Role of TGF-beta in survival of phagocytizing microglia: autocrine suppression of TNF-alpha production andoxidative stress. Exp. Neurobiol. 21, 151–157. 10.5607/en.2012.21.4.151 23319875 PMC3538179

[B61] Saijo K. Glass C. K. (2011). Microglial cell origin and phenotypes in health and disease. Nat. Rev. Immunol. 11, 775–787. 10.1038/nri3086 22025055

[B62] Schmechel D. E. Rakic P. (1979). Arrested proliferation of radial glial cells during midgestation in rhesus monkey. Nature 277, 303–305. 10.1038/277303a0 105294

[B63] Sequerra E. B. Costa M. R. Menezes J. R. L. Hedin-Pereira C. (2013). Adult neural stem cells: plastic or restricted neuronal fates? Development 140, 3303–3309. 10.1242/dev.093096 23900539

[B64] Sequerra E. B. Miyakoshi L. M. Fróes M. M. Menezes J. R. L. Hedin-Pereira C. (2010). Generation of glutamatergic neurons from postnatal and adult subventricular zone with pyramidal-like morphology. Cereb. Cortex 20, 2583–2591. 10.1093/cercor/bhq006 20154014

[B65] Seri B. García-Verdugo J. M. Collado-Morente L. McEwen B. S. Alvarez-Buylla A. (2004). Cell types, lineage and architecture of the germinal zone in the adult dentate gyrus. J. Comp. Neurol. 478, 359–378. 10.1002/cne.20288 15384070

[B66] Shigemoto-Mogami Y. Hoshikawa K. Goldman J. E. Sekino Y. Sato K. (2014). Microglia enhance neurogenesis and oligodendrogenesis in the early postnatal subventricular zone. J. Neurosci. 34, 2231–2243. 10.1523/jneurosci.1619-13.2014 24501362 PMC3913870

[B67] Shin W. H. Lee D. Y. Park K. W. Kim S. U. Yang M. S. Joe E. H.. (2004). Microglia expressing interleukin-13 undergo cell death and contribute to neuronal survival in vivo. Glia 46, 142–152. 10.1002/glia.10357 15042582

[B68] Sierra A. Encinas J. M. Deudero J. J. Chancey J. H. Enikolopov G. Overstreet-Wadiche L. S.. (2010). Microglia shape adult hippocampal neurogenesis through apoptosis-coupled phagocytosis. Cell Stem Cell 7, 483–495. 10.1016/j.stem.2010.08.014 20887954 PMC4008496

[B69] Streit W. J. (2001). Microglia and macrophages in the developing CNS. Neurotoxicology 22, 619–624. 10.1016/s0161-813x(01)00033-x 11770883

[B70] Su P. Zhang J. Zhao F. Aschner M. Chen J. Luo W. (2014). The interaction between microglia and neural stem/precursor cells. Brain Res. Bull. 109, 32–38. 10.1016/j.brainresbull.2014.09.005 25245208

[B71] Tang X. Falls D. L. Li X. Lane T. Luskin M. B. (2007). Antigen-retrieval procedure for bromodeoxyuridine immunolabeling with concurrent labeling of nuclear DNA and antigens damaged by HCl pretreatment. J. Neurosci. 27, 5837–5844. 10.1523/jneurosci.5048-06.2007 17537952 PMC6672250

[B72] Tavazoie M. Van der Veken L. Silva-Vargas V. Louissaint M. Colonna L. Zaidi B.. (2008). A specialized vascular niche for adult neural stem cells. Cell Stem Cell 3, 279–288. 10.1016/j.stem.2008.07.025 18786415 PMC6864413

[B73] Thanos S. Fischer D. Pavlidis M. Heiduschka P. Bodeutsch N. (2000). Glioanatomy assessed by cell-cell interactions and phagocytotic labelling. J. Neurosci. Methods 103, 39–50. 10.1016/s0165-0270(00)00294-6 11074094

[B74] Tsuda M. Masuda T. Tozaki-Saitoh H. Inoue K. (2013). Microglial regulation of neuropathic pain. J. Pharmacol. Sci. 121, 89–94. 10.1254/jphs.12r14cp 23337437

[B75] Ueno M. Fujita Y. Tanaka T. Nakamura Y. Kikuta J. Ishii M.. (2013). Layer V cortical neurons require microglia support for survival during postnatal development. Nat. Neurosci. 16, 543–551. 10.1038/nn.3358 23525041

[B76] Ventura R. E. Goldman J. E. (2007). Dorsal radial glia generate olfactory bulb interneurons in the postnatal murine brain. J. Neurosci. 27, 4297–4302. 10.1523/jneurosci.0399-07.2007 17442813 PMC6672317

[B77] Vilhardt F. (2005). Microglia: phagocyte and glia cell. Int. J. Biochem. Cell Biol. 37, 17–21. 10.1016/j.biocel.2004.06.010 15381143

[B78] Voigt T. (1989). Development of glial cells in the cerebral wall of ferrets: direct tracing of their transformation from radial glia into astrocytes. J. Comp. Neurol. 289, 74–88. 10.1002/cne.902890106 2808761

[B79] Walton N. M. Sutter B. M. Laywell E. D. Levkoff L. H. Kearns S. M. Marshall G. P.. (2006). Microglia instruct subventricular zone neurogenesis. Glia 54, 815–825. 10.1002/glia.20419 16977605

[B80] Yu Y. Ye R. D. (2014). Microglial a*β* receptors in Alzheimer’s disease. Cell. Mol. Neurobiol. 35, 71–83. 10.1007/s10571-014-0101-6 25149075 PMC11486233

[B81] Ziv Y. Ron N. Butovsky O. Landa G. Sudai E. Greenberg N.. (2006). Immune cells contribute to the maintenance of neurogenesis and spatial learning abilities in adulthood. Nat. Neurosci. 9, 268–275. 10.1038/nn1629 16415867

